# Delayed hyper-enhancement cardiac magnetic resonance imaging is more accurate than other noninvasive parameters in diagnosis of patients with endomyocardial biopsy positive cardiac amyloidosis

**DOI:** 10.1186/1532-429X-11-S1-O63

**Published:** 2009-01-28

**Authors:** Bethany A Austin, Scott D Flamm, E Rene Rodriguez, Carmela Tan, Randall C Starling, Milind Y Desai

**Affiliations:** Cleveland Clinic, Cleveland, OH USA

**Keywords:** Left Atrial, Endomyocardial Biopsy, Cardiac Amyloidosis, Myocardial Performance Index, York Heart Association Class

## Introduction

**Figure 1:**
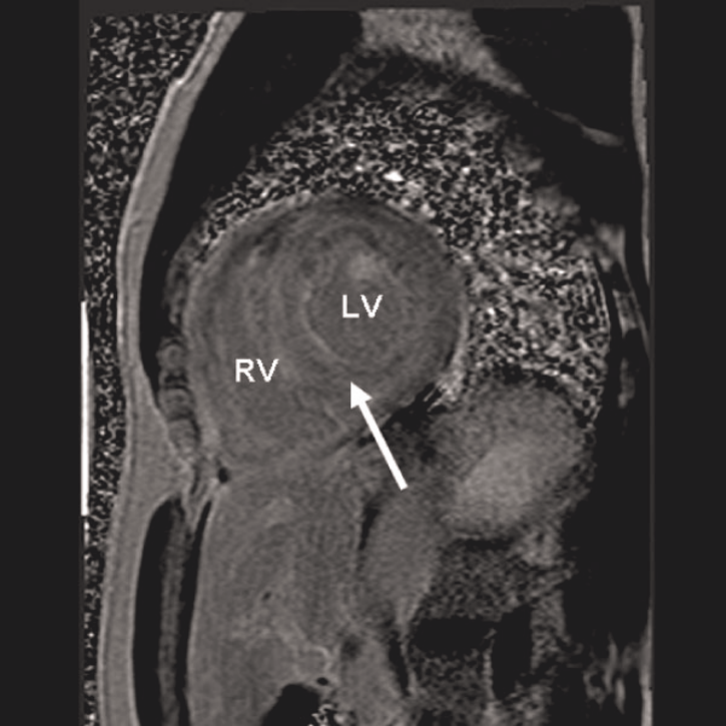
Figure 1

In patients with nonischemic cardiomyopathy and suspected cardiac amyloidosis (CA), endomyocardial biopsy (EMB) provides a definitive diagnosis. A multitude of noninvasive parameters, including electrocardiography (ECG) and transthoracic echocardiography (TTE) have been utilized as potential markers of CA. Recently, it has been demonstrated that, in patients with CA, delayed hyper-enhancement cardiac magnetic resonance (DHE-CMR) reveals a characteristic diffuse enhancement of the entire subendocardium (arrows) with extension into the neighboring myocardium (Figure 1).

## Purpose

We sought to determine the diagnostic accuracy of DHE-CMR, as compared to standard noninvasive parameters, in patients with suspected CA that underwent EMB.

## Methods

A total of 38 patients (mean age 62 ± 14 years, 71% men, 57% with New York Heart Association class > 2) with suspected CA underwent electrocardiography (ECG), TTE (including tissue Doppler), DHE-CMR (Siemens 1.5 T scanner, Erlangen, Germany) and EMB between 1/05 and 4/08. Low voltage on ECG was defined as sum of S wave in lead V1 + R wave in lead V5 or V6 < 15 mm. Measured TTE parameters included left atrial size, interventricular septal thickness, speckled appearance, E/A ratio, E/E' ratio, stage of diastology, deceleration time (msec) and myocardial performance index [(isovolumic contraction time + isovolumic relaxation time)/ejection time]. DHE-MR images were obtained in standard long and short axis orientations (covering the entire LV), after injection of Gadolinium dimenglumine using an inversion recovery spoiled gradient echo sequence: TE 4 msec, TR 8 msec, flip angle 30°, bandwidth 140 Hz/pixel, 23 k-space lines acquired every other RR-interval, field of view (varied from 228–330 in the x-direction and 260–330 in the y-direction) and matrix size (varied from 140–180 in the x-direction and 256 in the y-direction). CMR was considered positive in the presence of DHE of entire subendocardium with extension into the neighboring myocardium.

## Results

There were 17 each with EMB-positive CA and CMR-suspected CA. Using EMB as gold-standard, there was 2 false-positive and 2 false-negative CMR. Sensitivity, specificity, positive predictive value (PV) and negative PV of DHE-CMR in the diagnosis of CA were 88%, 90%, 88% and 90% respectively. Logistic regression analysis demonstrating the association between EMB-positive CA and various noninvasive parameters is shown in Table 1.

**Table 1:** Logistic univariate regression analysis testing the association between endomyocardial biopsy proven cardiac amyloidosis and various noninvasive imaging parameters

	Univariate		Multivariate	
Noninvasive imaging parameters	Wald χ^2^ Statistic	p-value	Wald χ^2^ Statistic	p-value
Carroll' Criteria on ECG	2.23	0.13		
Rahman's Criteria on ECG	0.60	0.40		
Dilated left atrium (> 20 cm^2^)	0.98	0.33		
Interventricular septal thickness	8.6	0.003	1.7	0.19
Left ventricular ejection fraction	0.04	0.83		
Speckled appearance on surface echocardiography	0.0	0.9		
Pseudonormal or restrictive physiology on Doppler echocardiography	2.1	0.15		
E/A ratio	1.9	0.17		
E/E' ratio ≥ 15	1.06	0.30		
Abnormal deceleration time (≤ 150 msec)	0.002	0.97		
Myocardial performance index	1.7	0.20		
Positive delayed hyper-enhancement cardiac magnetic resonance	16	< 0.001	9.6	0.002

## Conclusion

DHE-CMR is highly accurate in noninvasive diagnosis of EMB-positive CA as compared to standard ECG and TTE criteria. Incremental prognostic value of DHE-CMR in CA for clinical outcomes needs to be determined.

